# Supplementary material for: Nanocrystal superlattices as phonon-engineered solids and acoustic metamaterials
Source: Nat Commun. 2019 Sep 17;10:4236. doi: 10.1038/s41467-019-12305-3 (PMC6748911; doi:10.1038/s41467-019-12305-3)
Supplement: Supplementary file 1 — Supplementary Information [file 41467_2019_12305_MOESM1_ESM.pdf]

Supporting Information for: Nanocrystal Superlattices as Phonon-Engineered Solids and Acoustic Metamaterials

Nuri Yazdani[1], Maximilian Jansen[1], Deniz Bozyigit[1], Weyde M. M. Lin[1], Sebastian Volk[1], Olesya Yarema[1], Maksym Yarema[1], Fanni Juranyi[2], Sebastian D. Huber[3], Vanessa Wood[1]

[1] Materials and Device Engineering Group, Department of Information Technology and Electrical Engineering, ETH Zurich, Zurich CH-8092 Switzerland.

[2] Laboratory for Neutron Scattering and Imaging, Paul Scherrer Institute, CH-5232 Villigen PSI, Switzerland

[3] Institute for Theoretical Physics, ETH Zurich, 8093 Zürich, Switzerland.

Note 1 – Modelling of NC Superlattice Phonons

For the masses in our mass-spring model, we consider the nanocrystal core plus the mass of all ligands bound to its surface. We approximate truncated-octahedron PbS nanocrystals as sphere with radius *r*, so that the mass *m*(*r*) can be estimated as:

$$m(r) = 4/3\pi r^3 \rho_{PbS} + 8 \cdot 3^{1/3}\pi^{2/3} r^2 a_{PbS}{}^{-2} m_{Lig}, (1)$$

where ρ_{PbS} is the density of PbS, a_{PbS} is the PbS lattice constant, and m_{Lig} is the mass of one ligand. The number of ligand binding sites on the edges of the truncated-octahedron PbS nanocrystals is then

$$n_{FCC}(r) = (\pi/3)^{1/3} r/a_{PbS}, (2)$$

from which we can then calculate the expected TA and LA energies,

$$\hbar\omega_{TA} \sim 1.2\sqrt{\frac{k_{Lig}n(r)}{m(r)}} (3)$$

$$\hbar\omega_{LA} \sim 2\sqrt{\frac{k_{Lig}n(r)}{m(r)}},$$

We can use eq. 3 to estimate the phonon group velocity for a particular branch x, v_{gx},

$$v_{gx} \approx \frac{\omega_x}{k_x} = \frac{\omega_x a_{SL}}{\pi}, (4)$$

where a_{SL} is the lattice constant of the superlattice.

However, modelling the NC superlattice as a simple mass-spring network ignores the interaction of the vibrations of the NC cores and with the phonons of the superlattice. These interactions become stronger as the energies of the superlattice phonons approach those of the core lattice vibrations. In order to gauge the impact of these interactions on the phonon density of states, we develop a simplified 1-dimensional model (**Supplementary Figure 1**). Each NC is modelled as a 3-mass system, where the outer 2 smaller masses represent the mass of the Pb atoms along the edge of the truncated-octahedron PbS nanocrystals, $m_{Pb}(r)$, and the larger central mass represents the mass of the rest of the atoms in the NC, $m(r)$-$2m_{Pb}(r)$. The spring between the large and small masses is set such that the vibration between them has an energy $\sim\hbar\omega_X$. We compute the phonon density of states of this system, which for a chain of *N* nanocrystals, will consist of *N* vibrations at low energy corresponding to the superlattice vibrations, and 2*N* vibrations at higher energies corresponding to relative vibrations of the large and small masses. We compare the highest energy superlattice mode of the model, $\hbar\omega_{max}$, to our measured TA phonon energies from the relation $\hbar\omega_{TA} \sim 1.2(\hbar\omega_{max}/2)$, and the results are plotted in **Supplementary Figure 1** as a function of spring constant and nanocrystal radius,

for interactions with lattice vibrations of $\hbar\omega_X$= 10.5 meV and 6.0 meV (LA and TA phonon energies of bulk PbS). The results demonstrate that expected scaling of the superlattice phonons with spring constant or radius from the simple mass-spring model will be modified by the interaction with vibrations of NC cores, particularly relevant as the energy of the superlattice phonons approach the energies of the lattice vibrations.

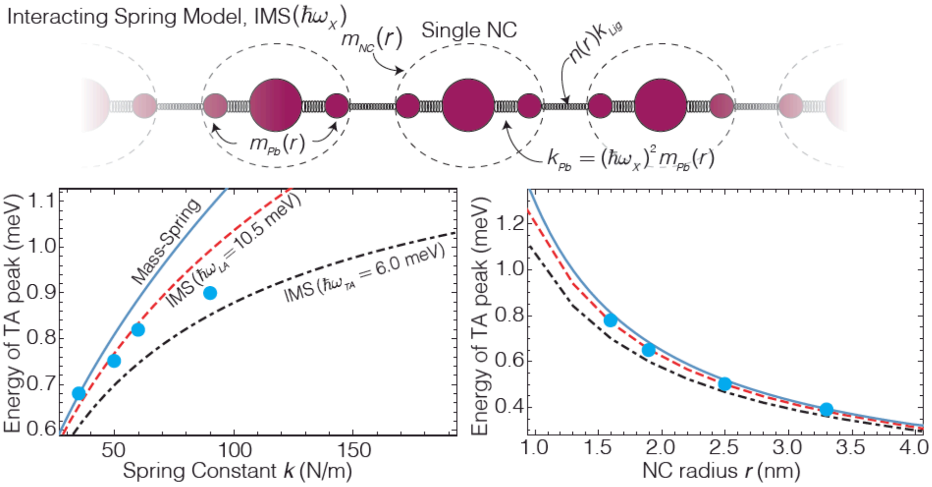

Supplementary Figure 1, **Interacting mass spring model** – Plots of the expected TA superlattice phonon energy as a function of ligand spring constant (with 1.6 nm NCs) and NC radius, assuming a simple mass-spring model (solid line) and including interactions with the LA and TA vibrations of the nanocrystal cores (dashed lines) calculated from the 1D model above.

Note 2 – Nanocrystal Samples

In **Supplementary Figure 2**, we plot the absorption spectra of the 7 as synthesized nanocrystal solutions used to prepare the NC-superlattice powders, measured on a Cary 60 UV-Vis system. The four samples of 1.6 nm radii used for the ligand series are comparable. The mean radii of the NCs are determined from the position of the first absorption peak using a well-established empirical model.[1]

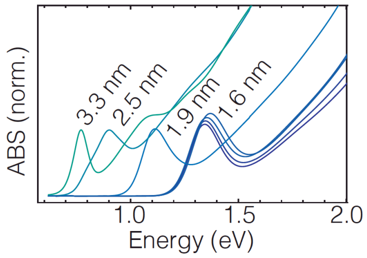

Supplementary Figure 2, Absorption spectra of as synthesized nanocrystal samples

Note 3– Extracting the density of states from INS data

As discussed in the main text, the integrated $g_{SL}(\omega)$, or number of inter-nanocrystal vibrations, is $3N_{NC}$-6, where N_{NC} is the total number of NCs in the NC-solid. In contrast, the integrated

density of lattice vibrations of the NC cores and ligands is $\int((g_{NC}(\omega)+g_{Lig}(\omega))$ is $N_{NC}(3N_A\text{-}6)$ where N_A is the number of atoms per NC including ligands. The number density of inter-NC vibrations is therefore ~N_A (100-1000) times smaller than number density $\int((g_{NC}(\omega)+g_{Lig}(\omega))$ meaning we are looking for very small features superimposed over a large background. Furthermore, from our modelling, we can expect $g_{SL}(\omega)$ to occur at energies $\hbar\omega$ ~ 0.2 – 2.0 meV, such that there will be an overlap between scattering from the $g_{SL}(\omega)$ with quasi-elastic scattering at low energy transfers resulting from reorientational motion of the surface terminating ligands. Extracting $g_{SL}(\omega)$ from INS thus requires careful and systematic subtraction of both the quasi-elastic scattering and $g_{NC}(\omega)$. In order to eliminate any sample-to-sample bias in the data analysis, we utilize a fully automated analysis procedure, applied identically to all measurements on all samples.

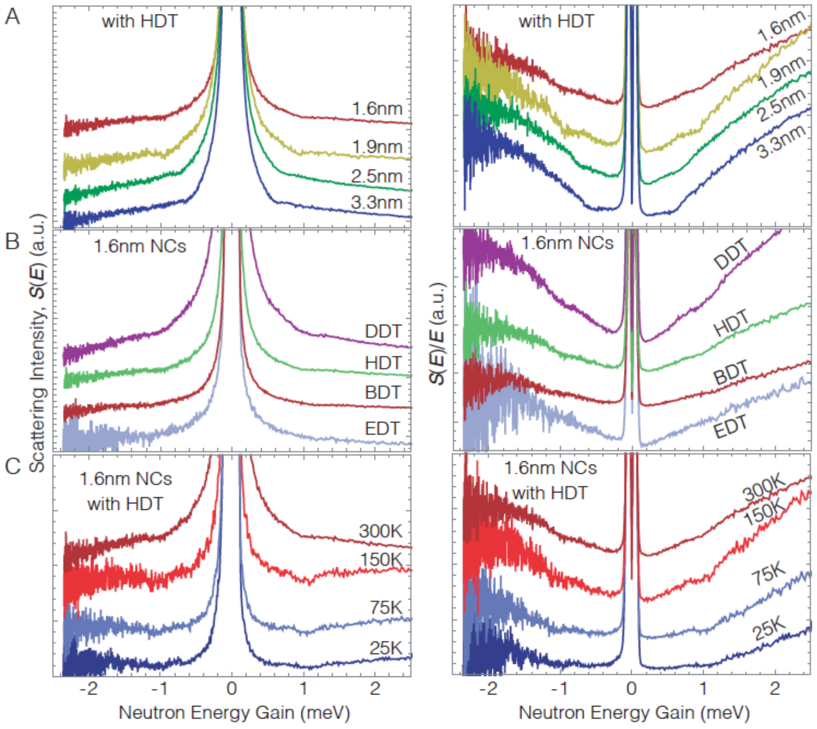

Supplementary Figure 3, Raw Inelastic Neutron Scattering data: (A) measured scattering, S(E) and S(E)/E for (A) NC-solids fabricated with HDT ligands and varying radii, (B) NC-solids fabricated with 1.6nm NCs and various ligands, and (C) 1.6 NC-solid fabricated with HDT ligands at several sample temperatures.

For each measurement, we obtain the scattering intensity as a function of energy (E) and momentum transfer (q), $S(q,E)$. We sum up the scattering over a q range from $1 < q < 3$ Å^{-1}, to obtain the inelastic scattering intensity as a function of energy transfer, $S(E)$. In **Supplementary Figure 3**, we plot the measured $S(E)$ for each of the samples presented in the main text.

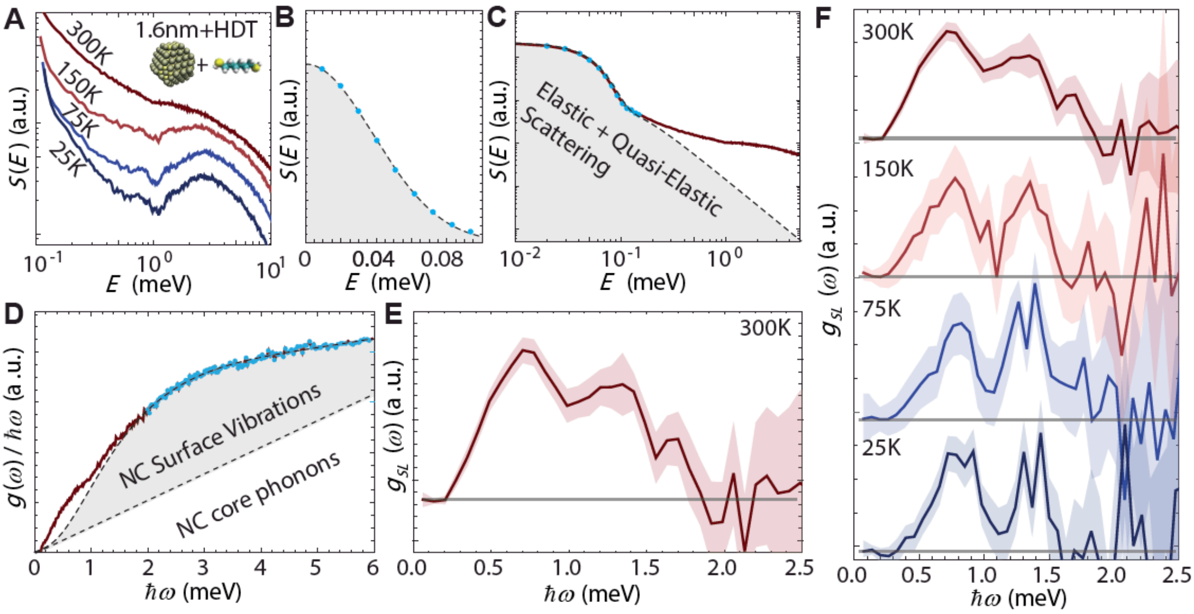

Supplementary Figure 4, Extracting the Density of Inter-NC Vibrations from Inelastic Neutron Scattering: (A) measured scattering, S(E), for a NC-solid fabricated with r = 1.6 nm NCs and HDT ligands at 300, 150, 75, and 25 K. The elastic peak (B) and quasi-elastic scattering (C) are fit and removed from S(E). The resulting g(ω) is then fit with a model for $g_{NC}(\omega)$ (D) which includes contributions from core and surface vibrations which when subtracted from g(ω), results in the $g_{SL}(\omega)$ shown in (E). The extracted $g_{SL}(\omega)$ for each temperature is then shown in (F).

The first step is removal of the elastic scattering and quasi elastic scattering from $S(E)$ (**Supplementary Figure 4A**). The elastic line can be sufficiently approximated by a Gaussian, N[E, σ], corresponding to the instrument resolution function, with a σ_R = 35.0 μeV (see **Supplementary Figure 4B**), determined by reference measurement on vanadium. Reorientational motion of the ligands cause quasi-elastic scattering which, in the simplest case, can be described by a Lorenzian lineshape centered at E = 0.[2] The instrument resolution broadens this further to a Voigt function, V[E, σ_R, γ]. We use the notation $S_{EQE}(E)$ for both contributions,

$$S_{EQE}(E) = A_N N[E, \sigma_R] + A_V V[E, \sigma_R, \gamma]. \quad (5)$$

We fit $S_{EQE}(E)$ to the measured $S(E)$, with A_N, A_V, and γ as fit parameters, using least squares minimization over an energy range of 0 - 175 μeV. An example of which is given in **Supplementary Figure 4C**. The total vibrational density of states of the sample, $g(\omega)$, can then be calculated as

$$g(\omega) = \frac{S(E) - S_{EQE}(E)}{n(E,T)}, \quad (6)$$

where $n(E,T)$ is the Bose occupation factor. In **Supplementary Figure 4D** we plot $g(\omega)/\hbar\omega$ for the 1.6nm/HDT NC-solid.

Finally, to extract $g_{SL}(\omega)$, we must determine and subtract $g_{NC}(\omega)$ from $g(\omega)$. We have previously characterized $g_{NC}(\omega)$ of PbS NCs via INS,[3] ab-initio molecular dynamics,[4] and inelastic x-ray scattering,[5] and have demonstrated that the partial density of vibrational states in the core of the NCs takes the form of the phonon density of states of bulk PbS, while the outer ~3 atomic layers of the NC admit vibrations localized about the NC surface with their own characteristic density of states. In particular, the Pb-rich [111] facets of the NCs admit a broad distribution of low energy (~3 meV) vibrations occurring below the transverse acoustic peak of bulk PbS (6 meV). We therefore choose to model the low energy (0-6 meV) portion of $g_{NC}(\omega)$ as a superposition of the transverse acoustic peak with a log-normal distribution (LN[μ,σ_{LN}]) of surface vibrations,

$$g_{NC}(\omega) = A_{TA}(\hbar\omega)^2 + A_S \mathrm{LN}[\mu, \sigma_{LN}], \quad \hbar\omega < 6 \text{ meV}. \quad (7)$$

We fit $g_{NC}(\omega)$ to the $g(\omega)$, with A_{TA}, A_S, μ, and σ_{LN} as fit parameters, using least squares minimization over an energy range of 2.0 – 6.0 meV. Such a fit for 1.6nm/HDT NC-solid is shown in **Supplementary Figure 4D**. The density of inter-NC vibrations can then be calculated as

$$g_{SL}(\omega) = g(\omega) - g_{NC}(\omega). \quad (8)$$

The resulting $g_{SL}(\omega)$ for the 1.6nm/HDT NC-solid is given in **Supplementary Figure 4E**.

The above procedure is fully automated, and identically applied to all measurements. While the choice of model for $g_{NC}(\omega)$ is ad-hoc, we note (i) it satisfies the requirement that $g_{NC}(0) = 0$, (ii) it provides an excellent goodness of fit for all samples, and (iii) the low energy features associated with $g_{SL}(\omega)$, although small, are nevertheless visible in $g(\omega)$ for all samples and for some samples in the raw data $S(E)$ (see **Supplementary Figure 4A**).

Quasi-elastic scattering, resulting from temperature activated processes, is strongly temperature dependent and, as shown in the main text, freezes out below ~175K. The inelastic scattering intensity from vibrational excitations, on the other hand, will change smoothly with temperature according to the thermal occupation factor $n(E,T)$. Our temperature dependent measurements thus provide us with a good sanity check for our data analysis procedure. The measured $S(E)$ for the 1.6nm/HDT NC-solid measured at 300, 150, 75, and 25 K are given in **Supplementary Figure 4A**, and the corresponding $g_{SL}(\omega)$ extracted at each temperature is reproduced from the main text in in **Supplementary Figure 4F**.

1. Yazdani, N. A., Bozyigit, D., Yarema, O., Yarema, M. & Wood, V. Hole Mobility in Nanocrystal Solids as a Function of Constituent Nanocrystal Size. *J. Phys. Chem. Lett.* **5,** 3522–3527 (2014).

2. Bee, M. *Quasielastic Neutron Scattering*. (1988).
3. Bozyigit, D. *et al.* Soft surfaces of nanomaterials enable strong phonon interactions. *Nature* **531,** 618–622 (2016).
4. Yazdani, N. *et al.* Tuning Electron–Phonon Interactions in Nanocrystals through Surface Termination. *Nano Lett.* **18,** 2233–2242 (2018).
5. Yazdani, N. *et al.* Measuring the Vibrational Density of States of Nanocrystal-Based Thin Films with Inelastic X-ray Scattering. *J. Phys. Chem. Lett.* **9,** 1561–1567 (2018).
